# Insights Into the Concept of Rheumatoid Arthritis Flare

**DOI:** 10.3389/fmed.2022.852220

**Published:** 2022-03-17

**Authors:** Emanuele Bozzalla-Cassione, Silvia Grignaschi, Blerina Xoxi, Terenzj Luvaro, Maria Immacolata Greco, Iolanda Mazzucchelli, Serena Bugatti, Carlomaurizio Montecucco, Antonio Manzo

**Affiliations:** Rheumatology and Translational Immunology Research Laboratories, Division of Rheumatology, IRCCS Policlinico San Matteo Foundation, University of Pavia, Pavia, Italy

**Keywords:** rheumatoid arthritis, flare, pathophysiology, clinical outcomes, composite disease activity indices

## Abstract

Identification of a pathological change in the course of systemic chronic immune-inflammatory diseases is key to delivering effective treatment strategies. In this context, one of the most compelling issues is the concept of flare. The multifaceted expression of disease activity in rheumatoid arthritis (RA) makes it challenging to provide an omni-comprehensive definition of flare, encompassing the pathology's different objective and subjective domains. Our incomplete understanding of the pathophysiological mechanisms underlying this process contributes to the partial comprehension of its potential clinical expression. This review focuses on the proposed pathophysiological processes underlying disease recrudescence in RA and the variable definitions adopted to capture flare in clinical practice through its objective, subjective, and temporal domains. Overall, what emerges is a complex landscape far from being unraveled.

## Introduction

Identifying a pathological change in systemic chronic immune-inflammatory diseases is key to effective treatment strategies. In this context, one of the most compelling issues is the concept of flare. Whilst this is an easily comprehensible disease dynamic in theoretical terms, its omnicomprehensive definition still faces several challenges in clinical practice. If, on the one hand, these challenges may derive from incomplete knowledge of pathogenic dynamics linked to clinical manifestations, on the other hand, they derive from the multiple domains that characterize the constructs “disease activity.” These include biological, subclinical, and subjective aspects of the pathology that might not be immediately measurable, not necessarily redundant, and whose relevance in the definition of the disease has been the object of continuous evolution.

A paradigmatic example of these concepts is rheumatoid arthritis (RA), which is frequently characterized by fluctuations in objective disease activity and subjective disease perception. RA hallmark characteristics had been identified, described, and collected within classification criteria by the ACR 1987 criteria (1). At that time, swollen joints only were considered pivotal in delineating the disease. Later, the EULAR/ACR 2010 criteria introduced tender joints as major defining findings of RA, recognizing elicited pain as a milestone characteristic of arthritis (2). Further, in the past few years, we have witnessed a breakthrough in the patient perspective, in the form of the so-called “patients reported outcomes,” gaining an increasing role in defining the disease itself and the goodness of treatment's response. From this short timeline, it is notable how the complexity of RA had progressively been unveiled. It has been recognized that RA embraces more subtle and not promptly measurable subjective domains besides the straightforward objective domains, all converging in the definition of the disease activity.

Due to the challenges discussed in the above paragraphs, numerous proxy definitions of RA flare (definable from a basic perspective, as the re-expression or enhanced expression of the disease pathogenic process) have been used in literature in the past years, according to the historical moment, the clinical context and the investigator's choice (Table 1).

**Table 1:** Rheumatoid arthritis flare definitions.

**Composite disease activity score-based flare**		
ΔDAS28 > 1.2 or > 0.6 if the final DAS28 ≥ 3.2		**(3–5, 8)**
ΔDAS28 > 1.2 or >0.6 if the initial DAS28 ≥ 3.2		**(6, 7)**
ΔDAS28 ≥ 1.2 or ≥ 0.6 if the initial DAS28 ≤ 3.2		**(9)**
ΔDAS28 > 0.6 and DAS28 > 2.6		**(10)**
ΔDAS28 > 1.2 or >0.6 if current DAS28 > 5.1		**(11–13)**
ΔDAS28 ≥ 0.6 and DAS28 > 3.2		**(14)**
ΔDAS28 > 1.2		**(15)**
ΔDAS ≥ 0.6 and DAS > 2.4 (any baseline DAS) or DAS > 2.4 from a previous DAS ≤ 2.4		**(16)**
DAS28-CRP ≥ 2.4		**(17)**
DAS28 > 3.2		**(18, 19)**
DAS28 > 2.6		**(20)**
DAS ≥ 1.6		**(21)**
DAS > 2.4 and/or SJC > 1		**(22, 23)**
**Patient-based flare**		
Δ ≥ 4.8-points in SF36-Bodily Pain score		**(9)**
RA Flare Questionnaire (no thresholds defined yet)		**(7)**
FLARE-RA questionnaire <2.3 (overall), <1.8 (arthritis subscale), and <3.8 (general symptoms subsale) rules out flare		**(24, 25)**
RAPID3 score > 2 SD above the baseline mean		**(26)**
RAPID3 > 4.27 (physician judgment) and > 4.33 (patient judgment)		**(27)**
RADAI5 > 4.5 (physician judgment) and > 4.7 (patient judgment)		**(27)**
“Over the last 3 months, did you experience symptoms suggestive of disease exacerbation?”		**(10)**
Complex clusterings of intense, unprovoked symptoms that defy self-management (not necessarily captured in joint counts or global VAS) that lead the patient to seek help		**(28)**
“During the past 6 months, have you had a flare in your rheumatoid arthritis?”		**(29)**
“Have you had any episode/episodes of tender and swollen joints?”		**(30)**
“Has your disease flared up since the last assessment?”		**(31)**
“Are you experiencing a flare of your RA at this time?” with a possible rating of severity and duration		**(6, 7, 32)**
**Physician-reported flare**		
Worsening of disease activity that required treatment beyond the permitted therapy based on investigator opinions		**(33)**
Worsening of signs and symptoms of sufficient intensity and duration to lead to a change in therapy		**(34)**
Any worsening of disease activity leading to initiation/change/increase of therapy or an expression such as “flare up,” “ongoing,” and “active” in the medical records		**(35)**
Recurrence of synovitis such that discontinuation of the protocol was considered necessary		**(36, 37)**
Investigator judgment of poorly tolerated flare		**(38)**
Doctor's intention to treat		**(39)**
**Combined flare definitions**		
**Objective**	**Subjective**	
**CDAI score > 10 or**	**Investigator's judgment of flare**	**(40)**
**ΔDAS28 ≥ 1.2 or ≥ 0.6 if final DAS28>3.2 OR**	**Investigator's judgment of flare**	**(41)**
**DAS28> 2.6 or inflammatory signs or**	**Inflammatory symptoms**	**(42)**
**Two of the following three: ΔDAS28 ≥ 1.2 and/or doubling of TJC and SJC and/or**	**Investigator's judgment of flare**	**(43)**

*DAS28, disease activity score on 28 joints; CRP, C-reactive protein; DAS, disease activity score; SJC, swollen joint count; SF-36, 36-Item Short Form Survey; RA, rheumatoid arthritis; RAPID3, routine assessment of patient index data 3; SD, standard deviation; RADAI5, Rheumatoid Arthritis Disease Activity Index-Five; VAS, visual analog scale; CDAI, clinical disease activity index; TJC, tender joint count*.

In this narrative review, we will provide an updated overview of the concept of flare in RA, focusing on the most recent studies exploring this process from a pathobiological perspective. We will then discuss the two main perspectives that have been currently pursued to translate the process into clinically applicable definitions: (1) perspectives based on composite indices, firmly rooted into the objective domains of the disease through pre-set algebraic thresholds, (2) perspectives based on patients or clinician judgment, thus primarily based on the subjective perception of the process and overcoming the intrinsic limit of composite indices sometimes at the expense of a lower standardization.

## Pathophysiology of RA Flare

**Figure 1:**
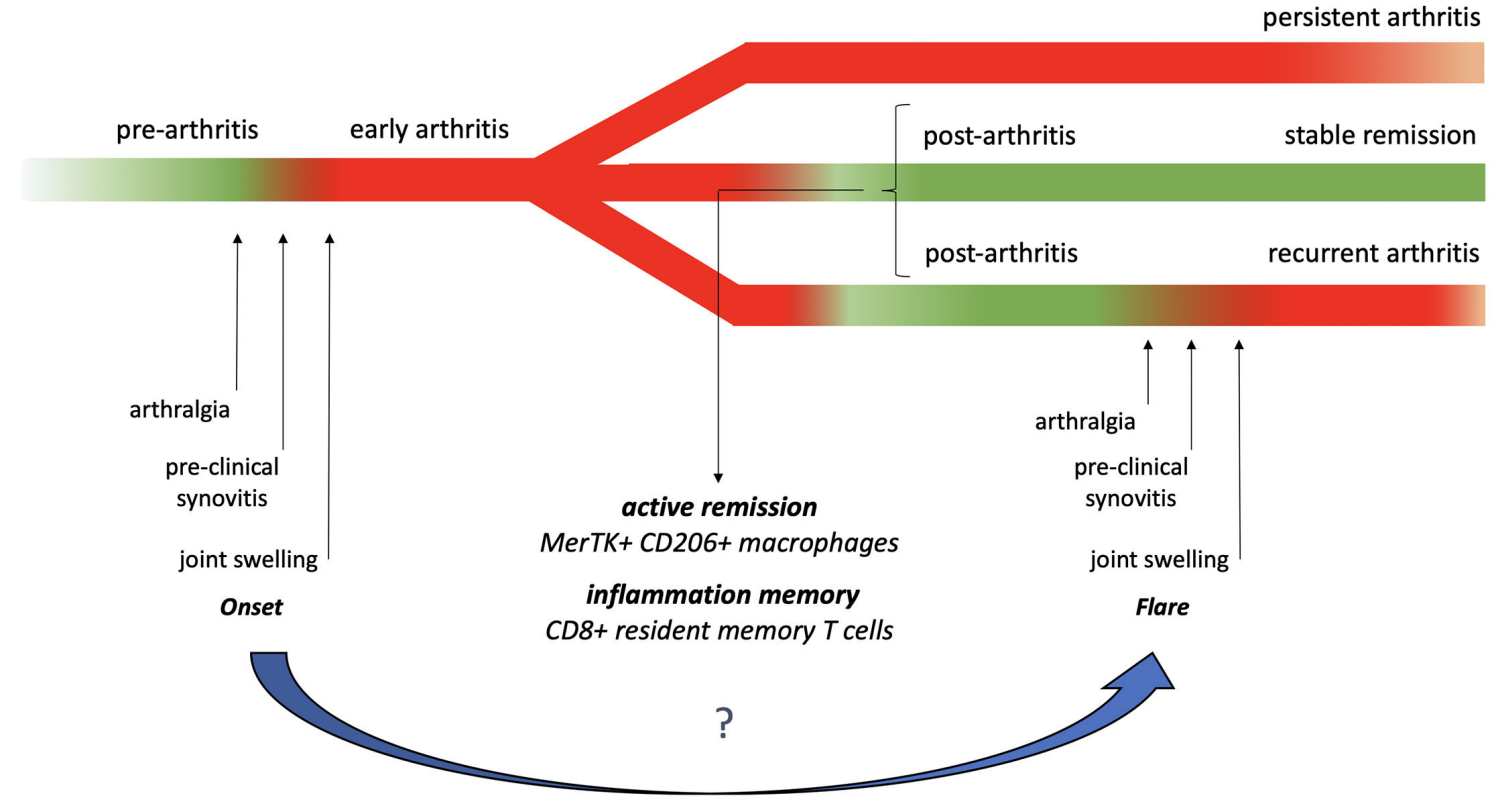
Rheumatoid arthritis activity natural history. Rheumatoid arthritis's natural history starts with pre-arthritis, where immunological and inflammatory changes occur in the absence of overt clinical manifestations. Following this phase, the process can evolve in overt joint swelling and in the typical clinical manifestations of RA. Patients may then experience a persistent state of active disease, reach and maintain remission, or experience a flare where a stepwise process resembling onset may occur. The mechanisms supporting the homeostasis in the post-arthritis remissions phase and the biologic dynamics of flare have not been fully clarified yet. The green and red sections of the diagram represent phases characterized by the absence or the presence of joint clinical manifestations, respectively.

Although the tissue and immune processes supporting active RA have been thoroughly investigated (44, 45), insights into the pathodynamics of remission and flare remain scarce. Currently, it is unclear whether the transition from arthritis remission to flare recapitulates the events involved in arthritis onset or whether the process is driven by different mechanisms in post-injured joints (Figure 1). Supporting the rationale of this question, various studies suggest the possibility that the remission status, rather than being a simple restitutio ad integrum, might be characterized by specific patho-biologic changes, including active processes in which the pathology is kept in check by regulatory mechanisms (balanced homeostasis) and inflammation memory traits.

Gene transcriptional profiling of peripheral blood mononuclear cells of children with polyarticular juvenile idiopathic arthritis in remission failed to demonstrate a return to normalcy, highlighting persistency of pro-inflammatory and anti-inflammatory genes networks, apparently keeping the pathologic process in balance (46). From a more peripheral (articular) perspective, evidence from experimental models and RA patients in remission demonstrated that arthritis resolution could be mechanistically based on enhanced induction of type 2 innate lymphoid cells (ILC2) by IL-9, which in turn elicits the activation of regulatory T cells (47). Resident eosinophils, consistently present in the synovia of patients with RA in remission, have been shown to promote arthritis resolution by secreting resolvins and switching synovial macrophages into the M2 phenotype (48). Only recently, Alivernini et al. (49) provided further evidence supporting the concept of active remission. A specific subset of tissue-resident macrophage has been identified in remission RA patients' synovia. In particular, MerTK positive, CD206 positive synovial tissue macrophages (STM) are up-regulated in the synovia of RA patients in remission with respect to active RA. These STM produce anti-inflammatory and resolving molecules acting on synovial fibroblasts and into the joint milieu, actively supporting the maintenance of joint homeostasis.

Focusing directly on the process of flare, Kuettel et al. explored the longitudinal associations between patient-reported flares and inflammatory dynamics on MRI. Pointing to an “outside-in” hypothesis, sequential analysis of inflammatory imaging changes in the hands showed a differential lesion pattern: synovitis and tenosynovitis increased early at flare onset, while bone marrow oedema evolved with delay and remained present for months (32). Two independent fascinating studies have recently pioneered in this specific RA phase through immune-pathologic analysis. Based on a longitudinal follow-up of patients with RA with sequential evaluations together with single-cell RNA sequencing of blood cells, Orange et al. (26) could identify a population of mesenchymal cells (PRIME, preinflammatory mesenchymal cells) exhibiting an increase in circulation just before flares of RA, but decreasing just after the appearance of symptoms. The expression analysis of PRIME cells identified a profile similar to that of synovial inflammatory sublining fibroblasts, suggesting a model involving the active migration of these cells into the flaring joint and their causative contribution to local inflammatory events. Chang et al. through the analysis of three different animal models of arthritis, have shown the possible long-term persistence of synovial resident memory T cells (Trm) in arthritic joints during remission. The same authors could also demonstrate the central role of CD8+ Trm in the maintenance of joint-specific memory in quiescent joints and in the mechanism of recurrent joint-specific flares (50). Whilst these data provide a potential immune-biologic explanation of the known trend of arthritis flare to recur preferentially in previously involved districts in human disease (51, 52), the mechanistic link between Trm activation in the joint and local homing of circulating PRIME cells remains to be clarified.

Beyond the systemic and “synoviocentric” perspectives on the pathology of RA flare, a particular emphasis has been given to the mechanisms of defective drainage and lymphatic flow (53). Evidence derived from elegant studies in the murine system and RA using indocyanine green dye and direct near-infrared imaging has actually shown the existence of potential defects in the exit process associated with active disease in flaring joints, a mechanism that expands the anatomical substrate potentially involved in the event of RA disease recurrence (54–56).

## Clinical Translation of the Concept of Flare: Composite Disease Activity Indices-Based Flare

Due to the lack of valid mechanistic biomarkers of flare, the goal of defining the process in clinical practice through quantitative and reliable approaches has led to the attempt to identify specific threshold adapting conventional indices of disease activity. Disease activity scores are widely used in clinical practice and as outcome measures in randomized clinical trials. All of them are built to embrace some of the most relevant defining aspects of RA activity, spanning through objective, subjective, and laboratory domains. However, their use and sensitivity to change have been validated limited to the improvement of the state of the disease, whether this was defined as achieving a designated level of relative improvement from baseline (American College of Rheumatology responses) (57), or as an improvement from baseline and specific state of activity of the disease in absolute terms [European League Against Rheumatism (EULAR) responses criteria] (58).

The OMERACT RA Flare workgroup provided a first working definition of flare in 2009: “A flare occurs with any worsening of disease activity that would, if persistent, in most cases lead to initiation or change of therapy; and a flare represents a cluster of symptoms of sufficient duration and intensity to require initiation, change or increase in therapy” (34). In 2013, van der Maas et al. tackled a more precise standardization providing specific thresholds to define minimal significant deviation capable of proving a substantial deterioration of the disease state, such as being called a flare. The proposed definition of flare is based on the DAS28 score and was obtained from the analysis of construct and criterion validity of previously proposed thresholds and minimal significant variations (3, 4, 11, 12, 15, 18, 59–61). DAS28-based flare is defined as DAS28 variation between two subsequent visits (Visit 1 and Visit 2) of more than 1.2 or more than 0.6 if the DAS28 was higher than or equal to 3.2 at the final visit (Visit 2) (sensitivity 63–78% and specificity 84–92%, using questions on disease activity worsening completed by patients and physicians as a gold standard's proxy) (5).

Before that, several unvalidated RA flare definitions have been used in clinical studies or proposed in the literature. These criteria vary considerably, ranging from physician-reported worsening to specific levels of change in core set variables or necessity to modify treatment (Table 1). However, the use of composite disease activity indices other than DAS28 in trials is limited to the work of Asai et al. (40), in which CDAI > 10 was used to define flare. Notably, an absolute value of DAS28 alone was frequently used when assessing flare in remission patients, where a variation of DAS28 may overestimate RA flare in patients fluctuating within the limits of remission (17, 19–22, 42). In some of these studies, to overcome the possibility of DAS28-based flare driven by subjective domains in the absence of objective synovitis, the additional or alternative presence of swollen joints was required (5, 21, 22, 61, 62). Indeed, it was observed that DAS28-based flare occurs more often than investigator-defined flare (1.7–7.3 times higher) (33) and, on the other hand, may miss patient-reported flare (PRF) (6). For this reason frequently the absolute threshold or minimal change in composite disease activity scores required to define flare were associated with or could be overcome by the investigator's judgment of flare (40, 41, 43).

Collectively, these data emphasize the potential limitations of the strict application of composite disease activity indices for the definition of flare in clinical practice (in particular in real life settings) and the relevance of the ongoing work of OMERACT to identify, define and standardize new domains, mainly based on patients' perception, to increase sensitivity and specificity of flare definition (34).

## Clinical Translation of the Concept of Flare: Patient-Based and Physician-Reported Flare

The physician judgment or the necessity of treatment modification has been applied in various works as the solely possible definition of flare (33, 34, 39). The rheumatologist's view can be actually considered as the only comprehensive tool to integrate information derived from the objective and laboratory parameters with the patient's perspective through the filter of an expert interpretation. The discordance between subjective and DAS28-based definitions of flare, as mentioned above, is however complicated by further discordance in different subjective definitions themselves. Indeed the agreement between patient-based and physician-reported flare (similarly to the agreement between patient- and DAS28-based flare) is significantly affected by the degree of disease activity. Among patients starting from a DAS28-defined remission status, a high agreement (κ's ≥ 0.73) was observed. In contrast, a progressively reducing agreement was observed in patients starting in low disease activity (κ's = 0.44) and moderate-high disease activity (κ's = 0.21–0.35) (6). This observation is well-reflected by the numerous subjective definitions of flare reported in the literature (Table 1).

Various complex validated questionnaires have been produced to assess patient-relevant domains. The OMERACT RA Flare workgroup recently validated the Rheumatoid Arthritis Flare Questionnaire (RA-FQ) (7) which encompasses pain, physical impairment, fatigue, stiffness, and participation, including those relevant domains identified in previous OMERACT works (63) and not covered by both Routine Assessment of Patient Index Data 3 (RAPID3) (64) and the Rheumatoid Arthritis Disease Activity Index-Five (RADAI5) (64); however, appropriate thresholds for determining RA flares have not yet been established. Differently, for RAPID3 and RADAI5, two broadly used self-report questionnaires in everyday practice both in the US and in Europe, cutoffs to identify flare based on physician and patient-reported perspectives have been proposed: 4.27 and 4.33 for RAPID3 and 4.5 and 4.7 for RADAI5, respectively (27).

The French-born FLARE-RA questionnaire is another possible tool to help the physician recognize flare from the patient's perspective (65). It has proved itself to be able to identify patients with fluctuating disease activity, especially in those patients with low disease activity or remission. Different cutoffs recognized to have good sensitivity and specificity have been proposed. Myasoedova et al. (24) identified a lower (for clinical detection) and upper (for therapeutic change) cutoff varying depending on the duration of disease. More recently, Aouad et al. (25) identified a clearer cutoff of 2.3 for the FLARE-RA general score, able to detect a patient “in flare” (above) vs. “not in flare” (below) over the past 3 months or since the last visit.

Modification of disease activity in RA is strongly related to pain, a subjective domain concretized with visual analog or numerical rating scales (9). Pain perception can be associated with tender joints or exist without elicitable joint pain. Indeed, pain has a complex biological background. The time spent in a chronic inflammatory state, such as RA, can affect different levels of the signaling cascade that modify perception and thresholds for pain (66, 67). Frequently, arthralgia is the first manifestation of RA even in the absence of objective synovial inflammatory processes or tender joints, like in pre-clinical arthralgia phases (68). Similarly, isolated arthralgia episodes frequently occur during the natural history of the disease and might be transient or prelude to recrudescence. Overall, the presence of pain, despite physician judgment, is a relevant domain identified by patients when defining flare. When we approach patient-based definitions of flare, it is critical to keep in mind that patient perception is partially modified by time spent with the disease. Experienced patients suggested that the longer you lived with the disease, the better you are at placing a worsening within the context of disease variability and not worrying about a flare (34).

Of interest, McWilliams et al. (9) recently proposed a new flare entity based primarily on pain and assessed by the SF36-bodily pain scale. They identified patients experiencing abrupt (primary) or progressive (incremental) pain flares as suggested by a minimal predetermined variation in the SF36-bodily pain scale. These exacerbations were discordant with DAS28 flare in 23 and 70% of cases, respectively. Despite a significant discordance rate between the pain and DAS28-based flare definitions, both were associated with a persistent increase in disability even after flare improvement.

Apart from validated questionnaires and scores, there are many domains not yet addressed by current assessment scales, which patients nevertheless recognize as essential aspects of disease activity. The intrinsic difficulty in measuring subjective domains has led many authors to evaluate the presence or absence of flare based on a simple anchor question considering the overall patient perspective: “have you experienced a flare since your last visit?” (66). This broad question provides an overarching summary of all the information that we miss to measure and that the patient recognizes as red flags of disease deterioration or recrudescence.

## Duration of Flare

Duration is one of the critical aspects that must be tackled to provide a solid definition of flare. Indeed, some exacerbations are short-lived (a “bad day”) and often managed with rest or non-pharmacological interventions. Others, more severe, may require clinical intervention (34). Fluctuation in disease activity is expected in the natural wax and weaning history of RA, and spontaneous resolution of a transient deterioration may be expected. Thus, differentiating flare from physiological fluctuations could avoid overtreatment strategies, which could be partially achieved by better understating the timing of flare.

Although the current criteria and definitions did not tackle systematically flare duration, some authors have addressed this point to better characterize flare.

The length of time spent by the patient in a flare state may span from a few days to several weeks in the current literature. Jacquemin et al. (31) reported that 79% of self-assessed flares were short flares (<3 days), while the remaining were persistent flares (more than 3 days) (31). The AMBRA trial (30) differentiated between transient reported flares (<14 days) and constant joint complaints when lasting for at least 1 year. In the DRESS study instead (8), the persistence of significant symptoms deterioration for more than 12 weeks was addressed as major flare, while shorter symptoms were considered short flares. The concept of time spent on flares, together with their frequency, is of tremendous importance. In fact, the length and frequency of flares are associated with radiographic progression and deterioration of physical function, an increase in CVD risk of 7% for each flare (considered to last 6 weeks), and a reduction in physical activity by a median of 1000 steps per day of flare, as recorded by connective activity trackers (31, 32, 35).

## Conclusion

The concept of flare in rheumatoid arthritis is blurred. The difficulty lies in the complexity of the multifaceted manifestations of rheumatoid arthritis, where subjective and objective domains converge in determining disease activity. The current criteria for the definition of flare are the first important step toward a better characterization to facilitate the recognition of this event in clinical research and trials. However, they still appear to lack those desirable omni-comprehensive capabilities for the routing application in real-life clinical practice. Challenges in this direction may derive not only from the intrinsic multi-dimensional nature of RA disease activity in individual patients but also from the potential heterogeneity of flare in different individuals. In particular, the predictable dynamic nature of the process of disease flare in RA might progress through various stages characterized by different expressiveness of objective and subjective domains (as in the case of the transition between pre-clinical and overt RA). The heterogeneous phatophysiological substrate of RA may delineate differences in the clinical expressiveness of flare in different disease subsets (for example, ACPA positive and ACPA negative RA). Finally, the objective and subjective expression of the flare process might be characterized by specific differences depending on the phase of the disease or its treatment protocol (early, late-stage, under treatment, or under drug-free conditions).

Collectively, this review points to the need for further research in this direction, a fundamental area of investigation that could turn out to be essential for improving patient monitoring, for the definition of new therapeutic targets, and for a deeper understanding of the pathophysiology of RA.

## Author Contributions

EB-C, SG, BX, TL, MG, IM, SB, CM, and AM contributed to the literature review and manuscript drafting. All authors contributed to the article and approved the submitted version.

## Conflict of Interest

The authors declare that the research was conducted in the absence of any commercial or financial relationships that could be construed as a potential conflict of interest.

## Publisher's Note

All claims expressed in this article are solely those of the authors and do not necessarily represent those of their affiliated organizations, or those of the publisher, the editors and the reviewers. Any product that may be evaluated in this article, or claim that may be made by its manufacturer, is not guaranteed or endorsed by the publisher.
